# Population pharmacokinetics and dose optimisation of colecalciferol in paediatric patients with chronic kidney disease

**DOI:** 10.1111/bcp.15064

**Published:** 2021-09-30

**Authors:** Mandy Wan, Bruce Green, Arpana Aprameya Iyengar, Nivedita Kamath, Hamsa V. Reddy, Jyoti Sharma, Jyoti Singhal, Susan Uthup, Sudha Ekambaram, Sumithra Selvam, Greta Rait, Rukshana Shroff, Jignesh P. Patel

**Affiliations:** ^1^ Pharmacy Department Guy's and St Thomas' NHS Foundation Trust, Evelina London Children's Hospital London UK; ^2^ Institute of Pharmaceutical Science King's College London London UK; ^3^ Model Answers R&D Brisbane Australia; ^4^ Department of Paediatric Nephrology St John's Medical College Hospital Bengaluru India; ^5^ Paediatric renal service unit, King Edward Memorial Hospital Pune India; ^6^ Department of Paediatric Nephrology Government Medical College Trivandrum India; ^7^ Department of Paediatric Mehta Multispecialty Hospital Chennai India; ^8^ Research Department of Primary Care and Population Health University of College London London UK; ^9^ Renal Unit UCL Great Ormond Street Hospital Institute of Child Health London UK; ^10^ Department of Haematological Medicine King's College Hospital Foundation NHS Trust London UK

**Keywords:** chronic kidney disease, colecalciferol, population pharmacokinetics

## Abstract

**Aims:**

The prevalence of vitamin D deficiency is high in children with chronic kidney disease (CKD). However, current dosing recommendations are based on limited pharmacokinetic (PK) data. This study aimed to develop a population PK model of colecalciferol that can be used to optimise colecalciferol dosing in this population.

**Methods:**

Data from 83 children with CKD were used to develop a population PK model using a nonlinear mixed effects modelling approach. Serum creatinine and type of kidney disease (glomerular *vs*. nonglomerular disease) were investigated as covariates, and optimal dosing was determined based on achieving and maintaining 25‐hydroxyvitamin D (25(OH)D) concentration of 30–48 ng/mL.

**Results:**

The time course of 25(OH)D concentrations was best described by a 1‐compartment model with the addition of a basal concentration parameter to reflect endogenous 25(OH)D production from diet and sun exposure. Colecalciferol showed wide between‐subject variability in its PK, with total body weight scaled allometrically the only covariate included in the model. Model‐based simulations showed that current dosing recommendations for colecalciferol can be optimised using a weight‐based dosing strategy.

**Conclusion:**

This is the first study to describe the population PK of colecalciferol in children with CKD. PK model informed dosing is expected to improve the attainment of target 25(OH)D concentrations, while minimising the risk of overdosing.

**What is already known about this subject**

- Vitamin D deficiency is prevalent in children with chronic kidney disease.
- Current dosing recommendations for vitamin D are based on limited pharmacokinetic data and the optimal dosing strategy is not known.

**What this study adds**

- This is the first population pharmacokinetic model describing the time‐course of 25‐hydroxyvitamin D 25(OH)D in children with chronic kidney disease receiving colecalciferol.
- A weigh‐based dosage regimen is proposed for achieving and maintaining 25(OH)D concentrations between 30–48 ng/mL.

## 1 INTRODUCTION

Vitamin D deficiency is widely prevalent in patients with chronic kidney disease (CKD), and contributes to abnormalities in calcium, phosphate and parathyroid hormone homeostasis with increasing recognition of its key role in the pathogenesis of CKD–mineral and bone disorder.^1^, ^2^, ^3^ International clinical practice guidelines provide consensus support for determining vitamin D status and correction of deficiency through vitamin D supplementation.^3^, ^4^

Circulating total 25‐hydroxyvitamin D (25(OH)D) is used clinically to assess an individual's vitamin D status. It reflects vitamin D supply from cutaneous biosynthesis and exogenous intake, and is not under any negative feedback control.^5^, ^6^, ^7^ Current CKD guidelines recommend initiation of vitamin D supplementation as for the general population,^4^, ^6^ with some expert panels recommending a target 25(OH)D concentration of at least 30 ng/mL.^3^, ^8^ Vitamin D supplementation is not without risks. While symptomatic vitamin D toxicity has been defined at 25(OH)D concentrations >100 ng/mL,^3^ population based cohort studies have suggested an association between increased mortality and 25(OH)D concentrations >48 ng/mL.^9^, ^10^ A more cautious supplementation approach is therefore adopted in children with reduced renal reserve including those with CKD.^3^

Despite its widespread use, the optimal dosing regimen of vitamin D supplementation required to correct and maintain adequate 25(OH)D concentrations in children is not known. Rich sampling pharmacokinetic (PK) studies are limited; the few studies in adults were conducted using large single doses of vitamin D, and studies involving children have focused on those with nutritional rickets.^11^, ^12^, ^13^, ^14^ Moreover, clinical studies have reported notable variations in individual response to vitamin D supplementation even when identical dosing regimens are compared in similar patient groups.^15^ These highlight the need for further PK data to guide dose optimisation in the paediatric population.

The Colecalciferol Supplementation in Children with Chronic Kidney Disease trial (C3 trial) was a prospective open‐label, multicentre, randomised controlled trial to test the efficacy of 3 different dosing regimens of colecalciferol (vitamin D_3_) for 12 months in children with CKD.^16^, ^17^ In the current study, these data were used to develop a population PK model to allow better understanding of colecalciferol PK, and through PK simulation, we propose dosing recommendations for achieving and maintaining 25(OH)D concentrations between 30–48 ng/mL in children with CKD.

## 2 METHODS

### 2.1 Patients and data collection

Data from the C3 trial were used for the population PK analysis.^16^, ^17^ Participating sites were located in India (between 8° and 18.5°N of the equator) and patients were recruited between December 2015 and September 2017. The trial enrolled children aged 1–18 years with CKD stages 2–4 with serum 25(OH)D concentrations <30 ng/mL. Children who received vitamin D preparations (including over‐the‐counter supplements) in the preceding 3 months, patients known to have nephrocalcinosis, or those with documented poor medication adherence were excluded.^16^, ^17^ The trial was registered under the Clinical Trials Registry of India (CTRI‐REF/2015/11/010180), received approval from the Institutional Ethics Committees of all participating centres, and was conducted in accordance with the ethical principles of the Declaration of Helsinki.^16^, ^17^

Children were randomised 1:1:1 to oral colecalciferol 3000 IU daily, 25 000 IU weekly or 100 000 IU monthly for 3 months (maximum of 3 courses) as part of the intensive replacement phase. Those who achieved 25(OH)D concentrations ≥ 30 ng/mL moved to the maintenance phase and received 1000 IU daily thereafter for up to 9 months. Data of children whose 25(OH)D concentrations fell below 30 ng/mL on the maintenance therapy but who continued to be followed up and prescribed the same colecalciferol product as in the trial were also included in this secondary analysis. Children received colecalciferol granules (D 360 granules, Torrent Pharmaceutical Limited, India) packaged and supplied by a central pharmacy.^16^

### 2.2 Analytics

Samples for 25(OH)D were drawn at assumed steady state. To minimise invasiveness, PK sampling was aligned with routine outpatient visits and occurred every 3 months. All samples were sent on the same day at ambient temperature to a central laboratory for analysis. Concentrations of total 25(OH)D were determined by isotope‐dilution liquid chromatography–tandem mass spectrometry (Waters Xevo TQ‐S, Waters, UK). The interassay coefficients of variation for total‐25(OH)D_2_ and total‐25(OH)D_3_ were <10%. All samples were within the limits of quantification (3.4–155.9 ng/mL).

### 2.3 Model development

Published data were used to guide model development.^18^, ^19^, ^20^, ^21^ In a model‐based meta‐analysis of PK data in healthy or osteoporotic adult subjects, a model with a central and a peripheral compartment (2‐compartment model) was found to best fit the data.^18^ In contrast, 1‐compartment models have been described in studies with sparse data.^19^, ^20^, ^21^, ^22^ Thus, both 1‐ and 2‐compartment models were tested using both untransformed and log‐transformed data. Body weight as the continuous covariate for apparent clearance and apparent volume parameters with allometric scaling was included a priori.^23^ Between‐subject variability terms were modelled and tested on each PK parameter using an exponential relationship as all PK parameters must be of positive values. Different error models were tested for estimation of residual variability. The selected base model was subsequently taken forward for covariate analyses by means of stepwise forward inclusion and backward elimination procedure. The specific covariates evaluated were those that had a mechanistic meaning: serum creatinine and type of kidney disease (glomerular *vs*. nonglomerular disease). Serum creatinine concentration was scaled by the expected sex‐ and age‐adjusted normal serum creatinine concentration as applied in other paediatric studies.^23^, ^24^, ^25^, ^26^, ^27^, ^28^

### 2.4 Model evaluation

Model evaluation was based on visual inspection of graphical diagnostics including predictions, residuals, as well as assessment of parameter estimates and precision of estimates. The comparison between 2 nested models (i.e., in covariate analyses) was performed based on the likelihood ratio test in which the difference in objective function value (OFV) is approximately χ^2^ distributed. Covariates were tested using the stepwise covariate modelling approach. Covariates were sequentially added to the base model and retained if a decrease in the OFV >3.875 was seen. A backwards elimination was then executed whereby all covariates that had been identified as significant were added to the base model and removed singularly to evaluate their continued relevance. An increase in the OFV of >6.635 (corresponding to *P*‐value <.01 in χ^2^ distribution with 1 degree of freedom) was required to retain the covariate in the final model.

Both the base and the final models were evaluated using nonparametric bootstrap analysis (*n =* 1000) to assess the robustness of the parameter estimates. The final model was also evaluated using prediction‐corrected visual predictive checks (pcVPC; *n =* 1000 simulations); the 5th, 50th and 95th prediction intervals, simulated from the posterior distribution of the final model parameter estimates, were overlaid with the 5th, 50th and 95th percentiles from the observed data. A well‐performing model would see the observed percentiles within the 90% confidence interval of the simulated predictions.

### 2.5 Simulations

Using the final model, 25(OH)D concentration–time profiles were simulated to assess current dosing recommendations (Table S1),^3^ and to evaluate alternative dosing regimens for achieving and maintaining target 25(OH)D concentrations between 30–48 ng/mL. A dataset of 10 000 hypothetical children was created based on demographic data from The Chronic Kidney Disease in Children Cohort Study (CKiD) cohort.^29^ Simulations were performed using subsets of the dataset with each containing 1000 children in each of the weight categories (12 to 20 kg, ≥20 to <40 kg and ≥40 to 70 kg) over a dosing period of 12 months. The simulations were based on children receiving colecalciferol at regular intervals and assuming 100% adherence to treatment. Consideration was applied to the safe upper limit and the no observed adverse effect level of 10 000 IU/d of colecalciferol as recommended by the Endocrine Society Practice Guidelines and the European Food Safety Authority.^8^, ^30^

### 2.6 Data analysis and software details

Population PK analyses were performed using NONMEM (version 7.4.3; ICON Development solutions, MD, USA) through the Pirana interface (version 2.9.6; Pirana Software & Consulting). Perl‐speaks‐NONMEM (PsN; version 4.9.0) was used for NONMEM run control. Data evaluation and graphic diagnostics were performed using R (version 3.6). Parameter estimation in NONMEM was performed using the First Order Conditional Estimation (FOCE) method (with interaction when appropriate).

## 3 RESULTS

The C3 trial recruited 90 children with CKD stages 2–4. There were 7 children who were lost to follow‐up after their baseline visits, leaving a total of 363 measurements of serum 25(OH)D concentrations from 83 children for population PK analysis. The median number of samples per child was 4 (range: 2–5). Demographic and clinical data of all children are presented in Table 1. The number of children receiving daily, weekly and monthly intensive regimens were 30, 27 and 26, respectively.

**TABLE 1:** Baseline characteristics of the study population

	All patients (*n =* 83)	
Age, y	9.4	(6.2–14)
Male, *n* (%)	58	(70%)
Asian, *n* (%)	83	(100%)
Anthropometry		
Weight, kg	23.9	(16–38)
Body mass index, kg/m^2^	15	(13.8–18.1)
Body surface area, m^2^	0.9	(0.7–1.2)
Type of renal disease, *n* (%)		
Glomerular disease	20	(24%)
Non‐glomerular disease	63	(76%)
Biochemistry		
25(OH)D, ng/mL	18.6	(13.4–23.4)
Creatinine, μmol/L	97.3	(66.3–153.9)
eGFR, ml/min per 1.73 m^2^	45.2	(29–63.6)
Corrected calcium, mmol/L	2.32	(2.25–2.5)
Phosphate, mmol/L	1.52	(1.42–1.74)
PTH, pg/mL	82	(53.1–174.2)

Data are presented as number with the percentage in parenthesis or as median and interquartile range in parenthesis. eGFR, estimated glomerular filtration rate (eGFR = k × height/serum creatinine, k = 0.413); 25(OH)D, 25‐hydroxyvitamin D; PTH, parathyroid hormone; Body surface area = square root (Height in cm × Weight in kg/3600); conversion factors for unit: serum 25(OH)D in ng/mL to nmol/L, × 2.496.

### 3.1 PK model development

25(OH)D concentration–time profiles were best described by a 1‐compartment model, with first‐order absorption and first‐order elimination on natural log‐transformed 25(OH)D concentrations. As none of the children received any vitamin D supplement at the start of the study, a basal concentration parameter, C_0_, was included reflecting endogenous 25(OH)D production from diet and sun exposure. Given that samples were taken to capture assumed steady‐state and 25(OH)D is known to have a long elimination half‐life, the absorption rate constant (K_a_) was fixed to 0.323/h based on published data.^18^ The best performing base model included a priori allometric weight scaling on apparent clearance and apparent volume centred on 24 kg (clearance for an individual = population value of clearance × (weight/24)^0.75^; volume of an individual = population value of volume × (weight/24)^1^; 24 kg is the median weight of the study population), between‐subject variability on clearance and basal concentration_,_ and an additive residual error on logarithmic transformed concentrations.

In covariate analysis, inclusion of serum creatinine on clearance did not improve the model, but the type of renal disease on clearance was found to improve the OFV by −4.83. However, this did not meet the statistical criteria for the backward elimination step, and therefore was not included in the final model. The mathematical representation of the final developed model is as follows:

$$\mathrm{CL}/F=\text{POPCL}\times {\left(\frac{\text{body weight}}{24}\right)}^{0.75}$$

$$V/F=\text{POPV}\times \left(\frac{\text{body weight}}{24}\right)$$

where CL/F is the apparent clearance, POPCL is the population estimate of clearance, V/F is the apparent volume of distribution and POPV is the population estimate of volume of distribution.

### 3.2 Final model

**FIGURE 1:**
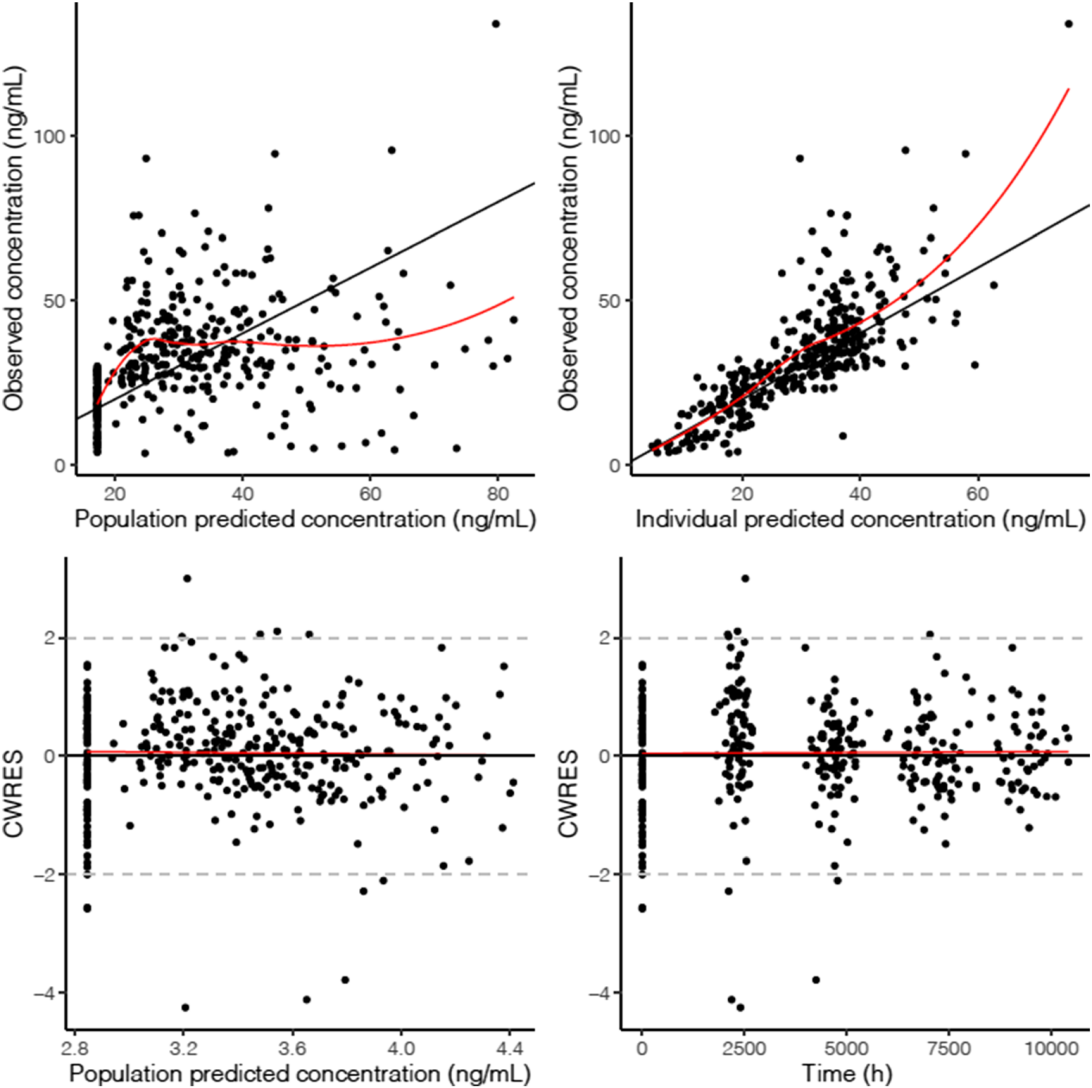
Goodness‐of‐fit plots for the final model. Top row: observations *vs*. population predictions, observations *vs*. individual predictions. Bottom row: conditional weighted residuals (CWRES) *vs*. population prediction, CWRES *vs*. time. Data have been back‐transformed for interpretation

Parameter estimates of the final PK model for colecalciferol‐25(OH)D are summarised in Table 2 (NONMEM code is provided in supplementary material). Eta shrinkage, which provides an assessment of the relevance of empirical bayes estimates‐based diagnostics, was 11.8% and 14.3% for the estimates of between‐subject variability of clearance and basal concentration, respectively. Goodness‐of‐fit diagnostics of the final model showed an adequate description of observed data (Figure 1).

**TABLE 2:** Parameter estimates from the final model

Parameter	Description	Estimate		Bootstrap	
		(% RSE)		Median	2.5–97.5 percentile
**Structural model**					
V/F, L	Typical value of apparent V	322	(31)	292	(199–572)
CL/F, L/h	Typical value of apparent CL	0.033	(23)	0.034	(0.011–0.043)
C_0_, ng/mL	Typical value of basal concentration	17.2	(6)	17.1	(15.4–19.2)
K_a,_ /h	Absorption rate constant	0.323	(fixed)	0.323	(fixed)
**Statistical model**					
ωCL/F, %CV	BSV of apparent CL	93.7	(42)	89.5	(63.5–162)
ωC_0_, %CV	BSV of C_0_	34.2	(32)	35.2	(23.6–44.7)
Residual, %CV	Residual variability	38.1	(19)	36.7	(29.8–43.5)

*Note:* Allometric scaling with exponents of 0.75 and 1 on apparent clearance (CL/F) and volume of distribution (V/F) were added a priori, standardised to 24 kg. BSV, between subject variability; CV%, coefficient of variation as a percentage; C_0_, basal 25(OH)D concentration; RSE, relative standard error.

**FIGURE 2:**
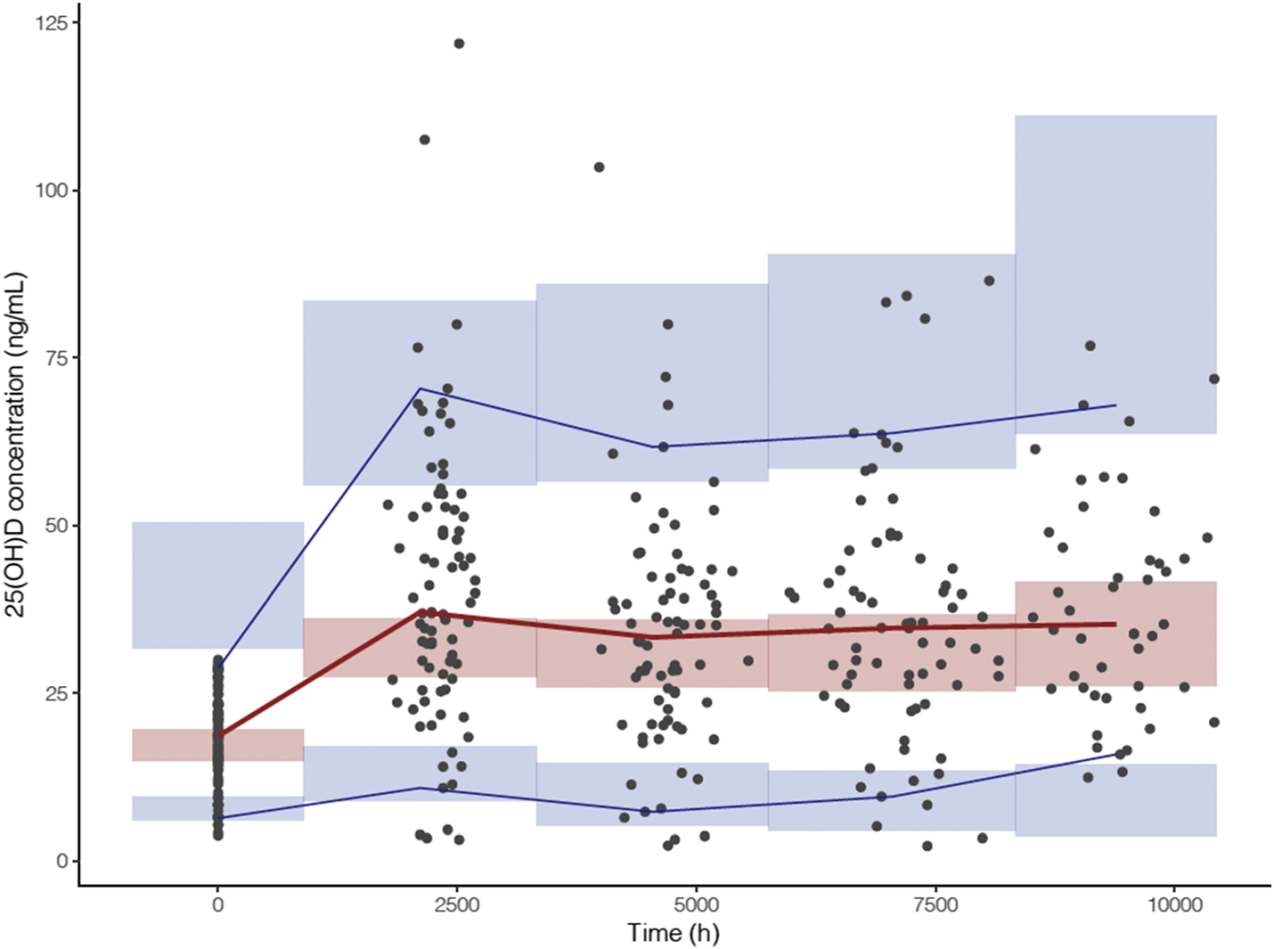
Predicted corrected visual predictive check of the final model. Lines represent the observed 25‐hydroxyvitamin D (25(OH)D) concentrations (5th, 50th and 95th percentiles) and the shaded areas represent the 95% confidence interval around the simulated 25(OH)D concentrations percentiles (5th, 50th and 95th percentiles). Black circles represent the observed data. *n* = 36 (9.9%) observed concentrations were outside the predicted range

The results from the 1000 bootstrap procedure demonstrated that the median and confidence intervals estimates were in alignment with estimates derived from the final model (Table 2). A pcVPC for the final model is presented in Figure 2; the similar distribution between simulated and observed data (with *n =* 36 [9.9%] observed concentrations outside of the predicted range) indicates that the final model developed was robust.

### 3.3 Simulation

**FIGURE 3:**
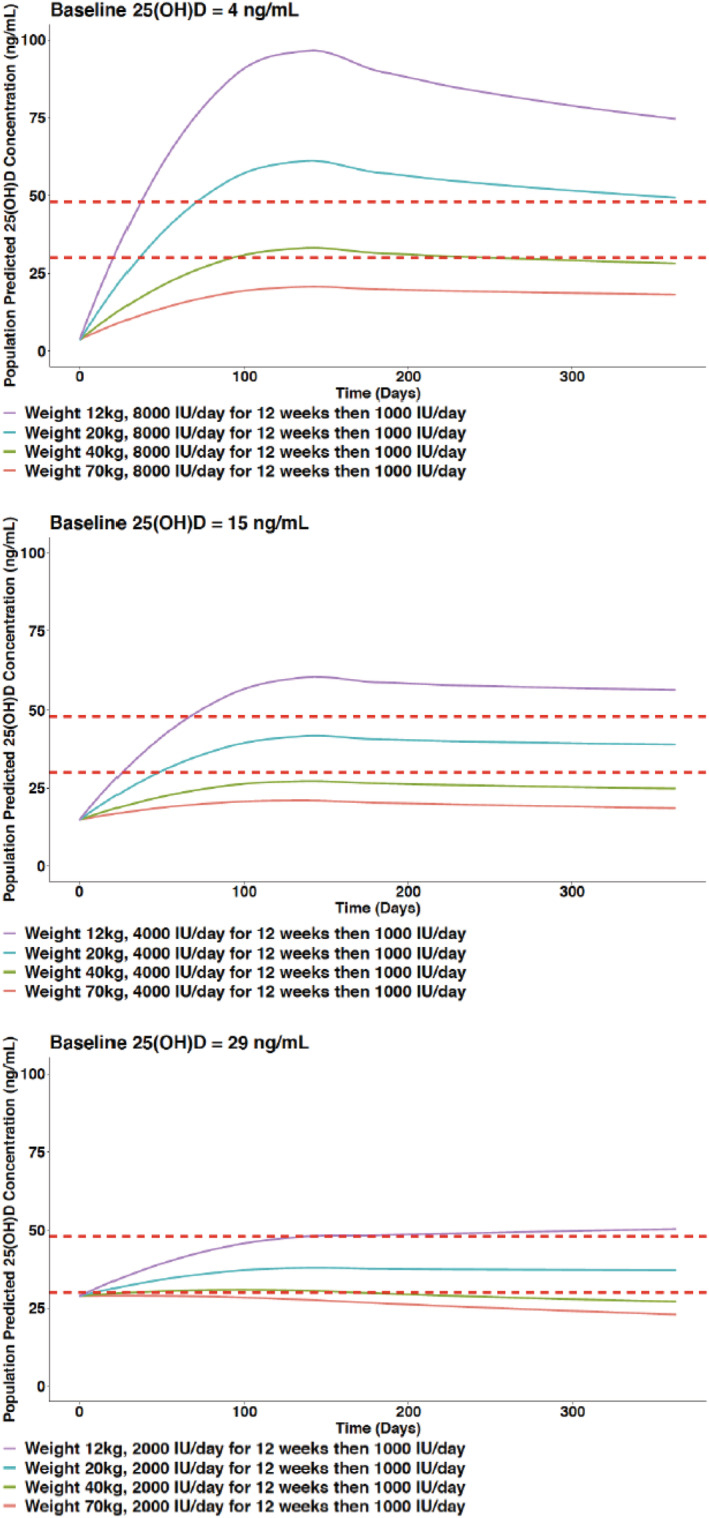
Simulated 25‐hydroxyvitamin D (25(OH)D) concentration–time profiles based on current dosing recommendations

**FIGURE 4:**
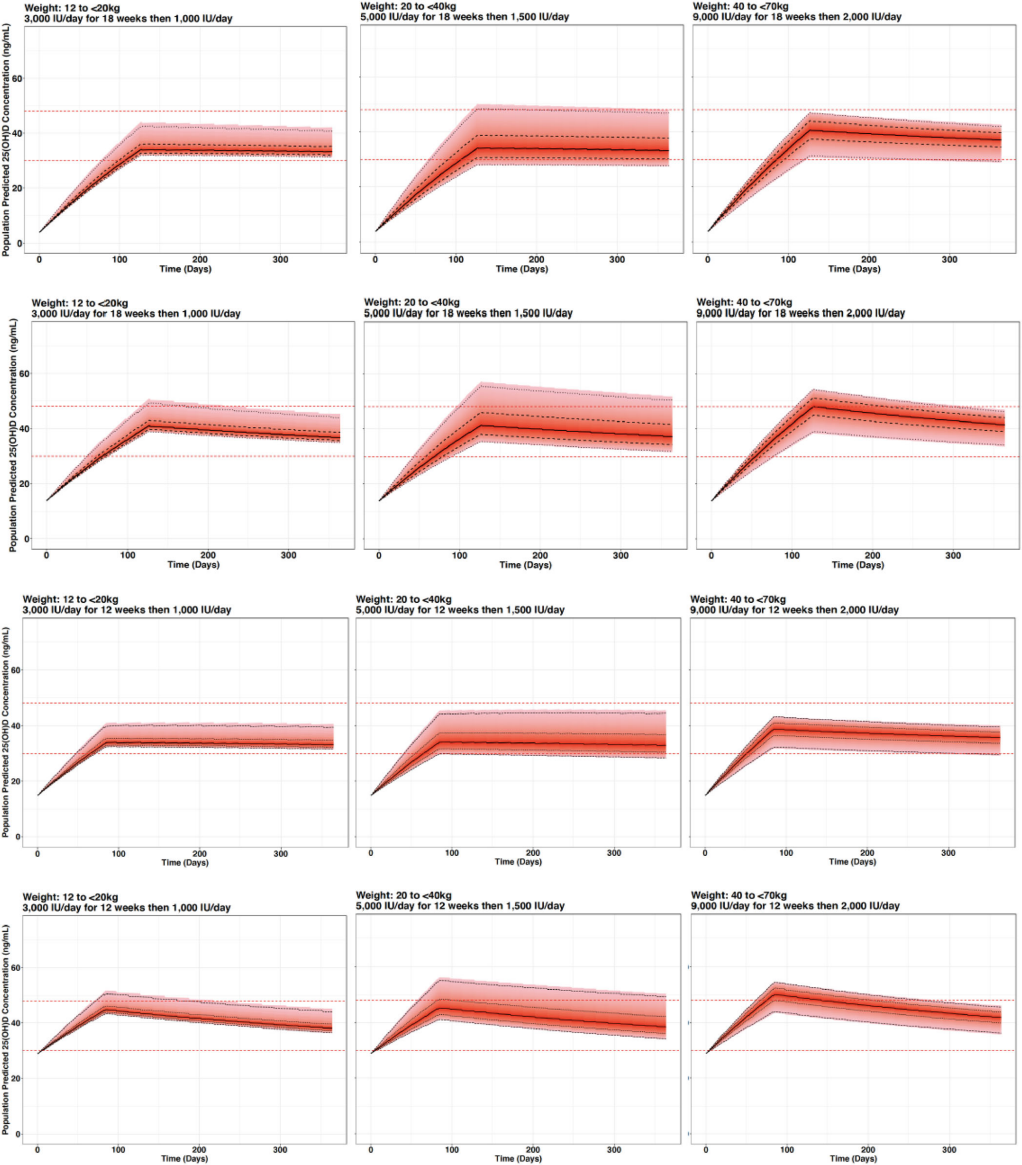
Simulated 25‐hydroxyvitamin D (25(OH)D) concentration–time profiles based on proposed weight‐based dosing regimens accounting for different baseline 25(OH)D concentrations (from top to bottom row: 4, 14, 15 and 29 ng/mL, respectively). The red horizontal dashed lines represent the target 25(OH)D concentration range of 30 and 48 ng/mL. Black lines represent the median (solid), 50% percentile interval (dashed) and 95% percentile interval (dotted)

**FIGURE 5:**
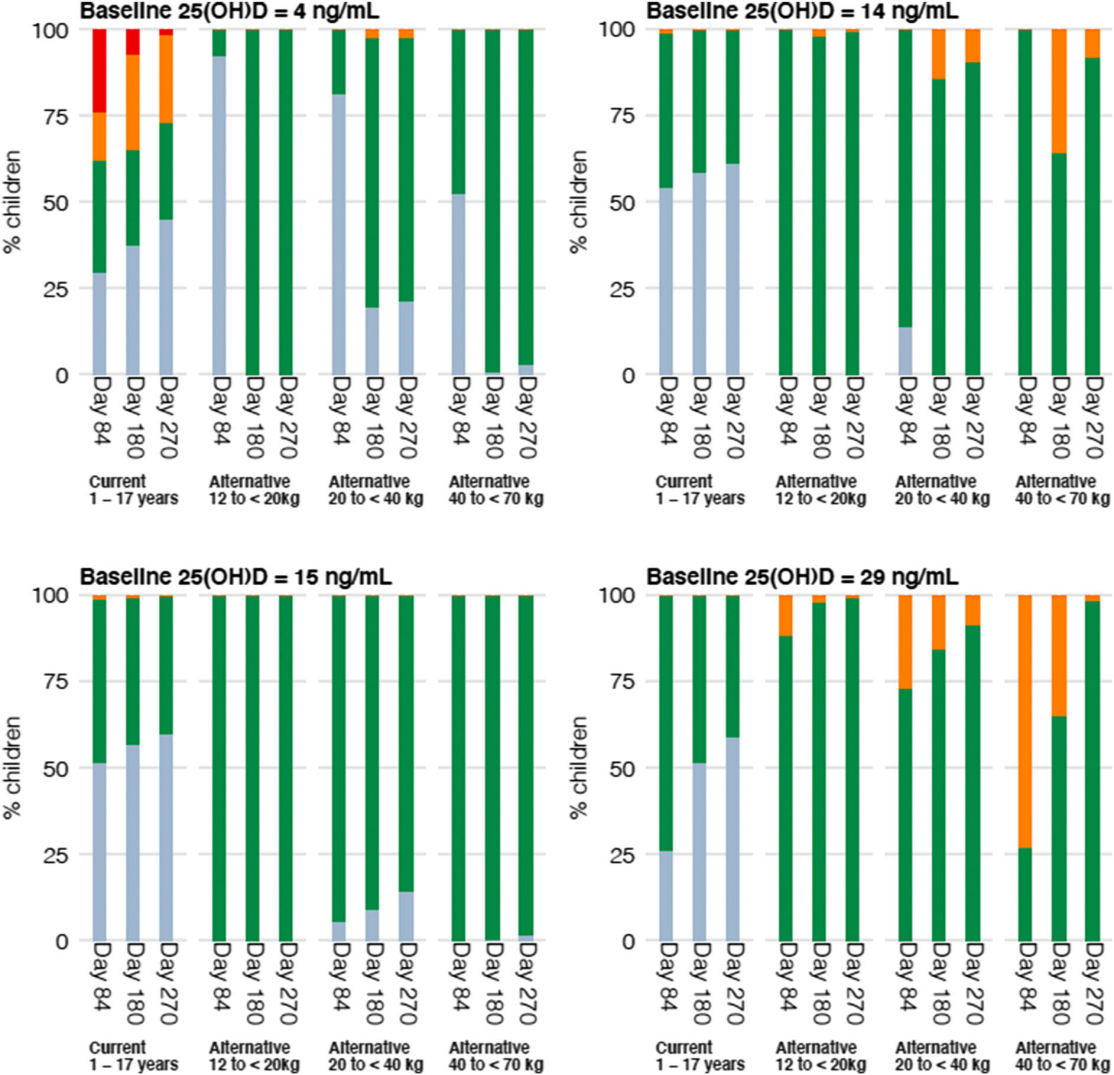
Simulated 25‐hydroxyvitamin D (25(OH)D) concentrations at different time points administered as current dosing recommendations and proposed weight‐based dosing regimens. The coloured bar represents the proportion of children with 25(OH)D concentrations < 30 ng/mL (blue), 30–47 ng/mL (green), 48–60 ng/mL (orange) and >60 ng/mL (red)

Simulations from the final model were generated to compare different dosing regimens. With current dosing recommendations, children with a body weight of 40–70 kg are unlikely to achieve the target 25(OH)D concentration range while children with lower body weight may achieve 25(OH)D concentrations exceeding 60 ng/mL (Figure 3). The simulated data (Figure 4) support a practical dosing regimen (Table 3) stratifying children by 3 weight bands (12 to <20 kg; 20 to <40 kg; 40 to <70 kg) and 2 baseline 25(OH)D concentration groups (<15 ng/mL; 15 to <30 ng/mL). The percentage of children achieving target 25(OH)D concentrations was greater with the proposed weight‐based regimens (Figure 5). While a proportion of children with a body weight of 40–70 kg may achieve 25(OH)D concentrations >48–60 ng/mL with the proposed weight‐based regimens (Figure 5), 25(OH)D concentrations were only transiently above the target range and substantially below the toxicity definition of 100 ng/mL (Figure 4).

**TABLE 3:** Proposed weight‐based dosing regimens

	Baseline 25‐hydroxyvitamin D concentration	
	< 15 ng/mL	15–30 ng/mL
**12 to <20 kg**	3000 IU/d for **18** wk then 1000 IU/d	3000 IU/d for **12** wk then 1000 IU/d
**20 to <40 kg**	5000 IU/d for **18** wk then 1500 IU/d	5000 IU/d for **12** wk then 1500 IU/d
**40 to <70 kg**	9000 IU/d for **18** wk then 2000 IU/d	9000 IU/d for **12** wk then 2000 IU/d

## 4 DISCUSSION

We have developed the first population PK model of oral colecalciferol using data from an randomised controlled trial in children with CKD. The PK model characterises the concentration–time course profiles of 25(OH)D and allows us to propose a weight‐based dosing regimen that would achieve and maintain 25(OH)D concentrations within the target range of 30–48 ng/mL.

These results provide an evidence‐based approach for colecalciferol dose optimisation in children with CKD.

Current dosing recommendations for colecalciferol in children in CKD are largely opinion based.^3^, ^31^ The recommendation from the Kidney Disease Improving Global Outcomes are nonspecific referring to dosing as per general population, while the European dosing recommendations are guided by baseline 25(OH)D concentration, where the same dose, irrespective of body size, is recommended for all children aged ≥1 year.^3^, ^4^ This is at odds with the widely accepted principle that physiological processes are scaled to body size, and could explain the large interindividual variation in dose–response observed in clinical practice, as well as possibly reflecting the general view that colecalciferol has a large therapeutic window. However, large observational studies in adults have shown a reverse J‐shaped association between increased all‐cause mortality and 25(OH)D concentrations >48 ng/mL.^9^, ^10^ Given children with pre‐existing renal impairment are at higher risk of developing hypercalcaemia and nephrocalcinosis, leading to a further deterioration in renal function,^3^ this provides a strong argument for a weight‐based dosing strategy in children with CKD.

Our finding for 25(OH)D clearance (0.0328 L/h standardised to a 24 kg individual [median weight of the studied population]; 0.0731 L/h standardised to a 70 kg individual) is in agreement with that reported by Foissac *et al*. in a cohort of children with human immunodeficiency virus; clearance was estimated to be 0.023 L/h (for comparison, the results has been scaled to a value for a 24 kg individual), which falls within the 95% confidence intervals of our estimate.^19^ The effect of CKD on 25(OH)D clearance remains unclear. While some observational studies have suggested impaired 25(OH)D clearance in CKD,^32^, ^33^ others hypothesised that CKD is a state of increased 25(OH)D clearance due to the induction of vitamin D 24‐hydroxylase by fibroblast growth factor‐23 which is elevated in patients with CKD.^34^

The high volume of distribution estimate in our study is perhaps to be expected given colecalciferol and 25(OH)D are both lipophilic molecules, but our estimated value is higher than those reported by Foissac *et al*. and in other adult studies.^19^, ^20^, ^21^ The differences in volume of distribution may be explained by differences in body composition of the study populations and/or differences in 25(OH)D protein binding. We acknowledge that there were insufficient data in our study to fully quantify a 2‐compartment model that may better describe the tissue distribution of colecalciferol and 25(OH)D. Earlier studies, although limited, have pointed to a biphasic distribution,^35^, ^36^, ^37^ and a 2‐compartment population PK model of colecalciferol has been described in only 1 meta‐analysis consisting of >5000 adult subjects.^18^ Nonetheless, model diagnostics of our model show a reasonable fit to guide dose optimisation, and our pcVPC showed good agreement with observations.

In our study, a notable difference with previous studies is the large between‐subject variability in 25(OH)D clearance. Compared to other studies which have also reported high between‐subject variability (60–65%) despite a relatively homogeneous population,^19^, ^20^, ^21^ our finding of 94% is likely to reflect an even more heterogenous cohort of patients with respect to 25(OH)D clearance. Although no covariate was identified as significant in our study, there is a strong biological plausibility that clearance could be affected in subjects with primary glomerular disease; our cohort included 20 children with glomerular disease, who compared to those with nonglomerular disease, required a longer intensive course of colecalciferol to achieve target 25(OH)D concentrations.^17^ In physiological conditions, vitamin D and its metabolites are transported in serum bound primarily (around 85–90%) to vitamin D binding protein (VDBP), and these complexes are filtered in the glomerulus and actively reabsorbed in the proximal renal tubule through megalin–cubulin mediated receptor endocytosis.^38^ However, this process is affected in tubular damage caused by proteinuria; previous studies have reported strong correlations between urinary excretion of VDBP and proteinuria, as well as higher excretion of urinary vitamin D in patients with nephrotic syndrome.^39^, ^40^, ^41^ The consequences would translate into an increase in 25(OH)D clearance as suggested by the forward inclusion step of our covariate analysis, but the small number of children with glomerular disease (*n =* 20; 24%) in our study probably explains the nonsignificant finding (during the backward elimination step) of the effect of glomerular disease on clearance. It should be noted that the influence of glomerular disease was modelled as a binary covariate in our study, and inclusion of urinary 25(OH)D data as a continuous measure in future studies may further help to explain between‐subject variation. A potential increase in the rate of 25(OH)D metabolism due to decreased protein binding resulting from loss of VDBP would also need to be considered.

Using the model developed, the simulations demonstrated that current dosing recommendations for colecalciferol can be optimised. To achieve and maintain a target 25(OH)D concentration of 30–48 ng/mL, simulations from our model support a weight‐based dosing strategy accounting for baseline 25(OH)D concentrations. The dosing simulations are in agreement with the results of a systematic review of individuals ≥10 years, which demonstrated that body weight is a significant predictor of change in 25(OH)D concentrations in individuals on vitamin D supplementation.^15^ This is further supported by a study in children with CKD, which demonstrated a significant positive association between dose per m^2^ body surface area per day and the change in 25(OH)D concentrations.^42^ Our simulations illustrate that while there is a large between‐subject variation in dose–response, the therapeutic window of 30–48 ng/mL allows us to propose a practical dosing strategy for use in clinical practice.

Our study has a few limitations that should be considered when interpreting the results. Samples for 25(OH)D concentrations were aligned with routine 3‐monthly outpatient visits to minimise patient burden and aimed at capturing steady state 25(OH)D concentrations. A study with sampling time points within the dosing intervals as well as different sampling time points between individuals would allow for better characterisation of colecalciferol PK, but the more intensive requirements would make such a study more challenging and limit patient numbers, especially in paediatrics.^23^ Our model also assumed constant endogenous production of 25(OH)D, although the effect of seasonal variation is likely to be minimal considering study sites are located between 8° and 18.5°N of the equator. Furthermore, all children were assumed to be adherent based on caregiver reported adherence measurement. It should be noted that data on Fitzpatrick skin phototype was not available and our cohort included only children of Asian ethnicity; although data is limited, recent study has suggested that 25(OH)D clearance may differ by race.^43^ We also acknowledge the discussions for and against the use of a priori allometric scaling in children, and that debate continues.^44^, ^45^ To further evaluate the PK of colecalciferol, research incorporating simultaneous measurements of colecalciferol, its metabolites including 1,25‐dihydroxyvitamin D and 24,25‐dihydroxyvitamin D, VDBP, total and unbound 25(OH)D (i.e. free‐25(OH)D) concentrations, along with data on different tissue concentrations would provide important information to guide future population PK analysis.

## 5 CONCLUSION

Using a population modelling approach, our study illustrates the limitation of current colecalciferol dosing recommendations in children with CKD and proposes a weight‐based dosing strategy for achieving and maintaining 25(OH)D concentrations in the target range.

## COMPETING INTEREST

No conflicts of interest to disclose.

## CONTRIBUTORS

M.W., G.R., R.S. and J.P. conceptualised this secondary analysis. A.I., N.K., H.R., J.S., J.S., S.U., S.E. and S.S. carried out the clinical trial data collection. M.W., B.G. and J.P. carried out the population PK modelling. M.W. drafted the initial manuscript. All authors critically reviewed and revised the manuscript.

## PATIENT CONSENT

All participants gave written informed consent prior to participation in the clinical trial.

## CLINICAL TRIAL REGISTRATION

Clinical Trials Registry of India; CTRI/2015/11/010180.

## Supporting information


**TABLE S1** European Society for Paediatric Nephrology recommendations for vitamin D therapy in children with chronic kidney disease.

## Data Availability

All data pertaining to the article are available in the article and in its supplementary material.
