# Supplementary material for: Population pharmacokinetics and dose optimisation of colecalciferol in paediatric patients with chronic kidney disease
Source: Br J Clin Pharmacol. 2021 Sep 30;88(3):1223–34. doi: 10.1111/bcp.15064 (PMC9291800; doi:10.1111/bcp.15064)
Supplement: Supplementary file 1 — TABLE S1 European Society for Paediatric Nephrology recommendations for vitamin D therapy in children with chronic kidney disease. [file BCP-88-1223-s001.docx]

**Supplementary material**

**Supplementary Table S1**

**Table S1: European Society for Paediatric Nephrology recommendations for vitamin D therapy in children with chronic kidney disease.**

| **Age** | **Serum 25-hydroxyvitamin D**  **(ng/mL)** | **Vitamin D supplementation dose (IU/day)** |
| --- | --- | --- |
| Intensive replacement phase (3 months) | | |
| >1 to <18 year | < 12 | 8,000 |
|  | 12 – 15 | 4,000 |
|  | 50 - 75 | 2,000 |
| Maintenance phase | | |
| >1 to <18 year | - | 1,000 – 2,000 based on CKD stage^a^ |

^a^ No further details are provided in the guideline in terms of which CKD stages.

**NONMEM model file with values set to final estimates**

$PROBLEM VITD_1CMT_LN(DV)

;----------------------------------

$INPUT ID ; subject identification number

DATE=DROP ; Date in mm/dd/yyy

TIME ; time of event in hr

AMT ; dose administered in micrograms

DVID ; dependent variable ID

MDV ; missing Dependent Variable,0=Not missing data value,1=missing data value

EVID ; event ID,0=observation,1=dose

AGE ; age in year

SEX ; sex,1=male,2=female

ETHN ; ethnicity,3=asian

WT ; weight in kg

HT ; height in cm

BMI ; body mass index

BSA ; body surface area

EGFR ; estimated glomerular filtration rate

CR ; serum creatinine in mg/dL

NKD ; 0=Non-glomerulus disease 1=Glomerulus disease

DV ; dependent variable, natural log of observed plasma concentration, ng/mL

ADCR ; age and sex adjusted serum creatinine

;----------------------------------

$DATA C3_NONMEM_FILE_CR_LNDV.csv IGNORE=I ;natural log transformed dataset

;----------------------------------

$SUBROUTINE ADVAN2 TRANS2

;----------------------------------

$PK

TVV = THETA(1)*(WT/24)

TVCL = THETA(2)*((WT/24)**0.75)

TVBC = THETA(3)

TVKA = THETA(4)

V = TVV

CL = TVCL*EXP(ETA(1))

BC = TVBC*EXP(ETA(2))

KA = TVKA

A_0(2)=BC*V ; amount of 25OHD in central compartment at time zero

;----------------------------------

S2 = V

;----------------------------------

$ERROR

IPRED = A(2)/V

IF(IPRED.GT.0) IPRED = LOG(A(2)/V)

IRES = DV-IPRED

Y=IPRED+EPS(1)

;----------------------------------

$THETA

(0, 322) ; V/F (L)

(0, 0.0328) ; CL/F (L/hr)

(0, 17.2) ; BC (ng/ml) (mean 25OHD = 18 ng/ml from dataset)

0.323 FIX ; KA (hr-1) (Ka from Ocampo-Pelland et al)

;----------------------------------

$OMEGA BLOCK(2)

0.878

-0.273 0.117

;----------------------------------

$SIGMA

0.145

;----------------------------------

$ESTIMATION METHOD=1 MAXEVAL=9999 PRINT=5 SIG=3

;----------------------------------

$COVARIANCE PRINT=E

;----------------------------------

$TABLE ID TIME AMT DVID DV MDV EVID AGE SEX ETHN WT HT BMI BSA EGFR CR NKD ADCR CL V KA

BC ETA1 ETA2 IPRED IRES CWRES ONEHEADER NOPRINT FILE=run308.fit

;----------------------------------

$TABLE ID TIME MDV EVID IPRED IRES CWRES ETA1 ETA2 NOPRINT ONEHEADER FILE=sdtab308

;----------------------------------

$TABLE ID CL V KA BC ETA1 ETA2 NOPRINT NOAPPEND ONEHEADER FILE=patab308

;----------------------------------

$TABLE ID AGE WT HT BMI BSA EGFR CR ADRC NOPRINT NOAPPEND ONEHEADER FILE=cotab308

;----------------------------------

$TABLE ID SEX ETHN NKD NOPRINT NOAPPEND ONEHEADER FILE=catab308
